# Molecular detection and characterization of haemoparasites in captive tigers (*Panthera tigris*) from Thailand

**DOI:** 10.1016/j.crpvbd.2025.100249

**Published:** 2025-02-18

**Authors:** Tanasak Changbunjong, Tatiyanuch Chamsai, Siriporn Tangsudjai, Nareerat Sangkachai, Chalisa Mongkolphan, Luxsana Prasittichai, Tanawat Chaiphongpachara

**Affiliations:** a Department of Pre-Clinic and Applied Animal Science, Faculty of Veterinary Science, Mahidol University, Nakhon Pathom, 73170, Thailand; b The Monitoring and Surveillance Center for Zoonotic Diseases in Wildlife and Exotic Animals (MoZWE), Faculty of Veterinary Science, Mahidol University, Nakhon Pathom, 73170, Thailand; c Protected Area Regional Office 3 (Ban Pong), Department of National Parks Wildlife and Plant Conservation, Ratchaburi, 70110, Thailand; d Department of Public Health and Health Promotion, College of Allied Health Sciences, Suan Sunandha Rajabhat University, Samut Songkhram, 75000, Thailand

**Keywords:** Molecular identification, *Rhipicephalus sanguineus*, *Ehrlichia canis*, *Hepatozoon canis*, *Hepatozoon felis*, Tick, Wildlife

## Abstract

Haemoparasites of the genera *Ehrlichia*, *Hepatozoon*, and *Babesia*, which are known tick-borne pathogens, infect a wide variety of domestic and wild animals. The aim of this study was to conduct a comprehensive molecular detection and characterization of haemoparasites in captive tigers (*Panthera tigris*) at a wildlife center in Thailand. From multiplex PCR results, haemoparasites were detected in the blood of 12 out of 17 tigers (70.6%), including 4 males and 8 females. Ten tigers (58.8%) were infected with *Ehrlichia canis*, one (5.9%) was co-infected with *Hepatozoon* sp. and *E. canis*, and another (5.9%) was infected solely with *Hepatozoon* sp. No infection with *Babesia* spp. was found. Nucleotide sequence analyses of the VirB9 protein gene sequence of *E. canis* and the 18S rRNA gene sequences of *Hepatozoon* spp. revealed high levels of genetic similarity with GenBank reference sequences. The *Hepatozoon* spp. sequence from the co-infected tiger showed 98.1–99.9% similarity with *Hepatozoon canis*, while another sequence showed a 97.6–99.7% match with *Hepatozoon felis*. The detection of these parasites underscores the complex interactions and dynamics of disease transmission that exist within captive environments, highlighting the need for preventive measures. Therefore, appropriate steps should be taken to control ectoparasites and manage domestic animals within wildlife centers to minimize the risk of infection.

## Highlights

• Haemoparasites were detected in 17 captive tigers (70.6%) examined at a wildlife breeding center, Thailand.
• *Ehrlichia canis* was detected in captive tigers for the first time.
• Molecular analysis confirmed infections with *Hepatozoon felis* and *Hepatozoon canis.*

## Graphical abstract

**Figure:**
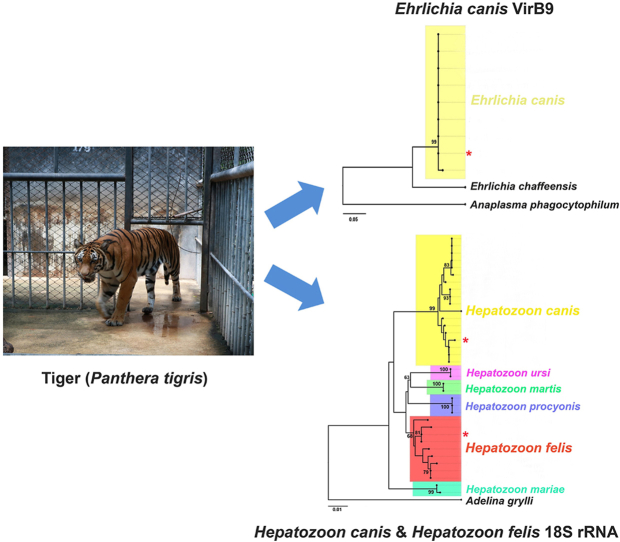

## 1 Introduction

The tiger (*Panthera tigris*) belongs to the family Felidae, which includes other large cat species such as lions, leopards, and jaguars (Nowell and Jackson, 1996). Within this family, the genus *Panthera* is distinguished by its members’ ability to roar, a characteristic trait that sets them apart from other felids (Lamberski, 2015). Tigers are the largest members of this genus and are crucial apex predators in the ecosystems they inhabit. They are found primarily in Asia, with their distribution extending from the Russian Far East to Southeast Asia and parts of China and India (Nowell and Jackson, 1996; Seidensticker, 2010). In natural forests, tigers play a critical role in maintaining biodiversity by regulating prey populations such as large deer and wild cattle, which helps prevent overgrazing and promotes the growth of plant species (Seidensticker, 2010; Ripple et al., 2014). The presence of tigers also creates a cascade effect throughout the food chain, helping to maintain the populations of smaller predators and scavengers that depend on prey availability.

In Thailand, outside natural forest habitats, the wildlife breeding centers operated by the Department of National Parks, Wildlife and Plant Conservation, play a crucial role in the conservation of rare and nearly extinct species (Jangtarwan et al., 2020; Pumpitakkul et al., 2023). These centers provide a controlled environment for breeding programmes, which are essential for preserving genetic diversity and supporting the recovery of endangered species. As tiger populations face significant threats such as habitat loss, poaching, illegal trade, and human-wildlife conflict, these breeding centers have become vital refuges (Erzinçlioğlu et al., 2024). In addition to breeding, some centers also quarantine and rehabilitate rescued animals that have been victims of wildlife crime (Tangsudjai et al., 2010). However, the management of captive tigers also presents challenges, particularly concerning infectious diseases. In confined environments, tigers are at risk of contracting pathogens such as canine distemper virus, severe acute respiratory syndrome coronavirus 2 (SARS-CoV2), feline leukemia virus (FeLV), *Toxoplasma gondii*, and haemoparasites (Thiangtum et al., 2006; Tangsudjai et al., 2010; Sangkachai et al., 2022; Gilbert et al., 2023). These diseases can spread more easily in captivity, where tigers are in close proximity to each other, and can pose a serious threat to their health.

Haemoparasites of the genera *Ehrlichia*, *Hepatozoon*, and *Babesia* are tick-borne pathogens that infect a wide variety of domestic and wild animals (Penzhorn, 2006; Pereira et al., 2018; Aziz et al., 2022; de la Fuente et al., 2023; Diarra et al., 2023). *Ehrlichia*, a genus of the family *Anaplasmataceae*, is an obligate intracellular bacterium transmitted primarily through tick bites. *Ehrlichia canis*, the causative agent of canine monocytic ehrlichiosis, has been reported in both domestic dogs and wild carnivores, although clinical cases in wild carnivores remain relatively rare (André, 2018). In wild dogs, *E. canis* has been observed to cause clinical signs similar to those seen in domestic dogs, including anorexia, depression, fever, and a range of hematological abnormalities such as anemia, leucopenia, and thrombocytopenia (André, 2018). *Hepatozoon* spp., apicomplexan protozoans belonging to the family Hepatozoidae, are primarily transmitted through the ingestion of infected ticks. In many cases, *Hepatozoon* spp. infections in wild animals can be subclinical. However, in some instances, the infection manifests in mild to severe forms, depending on the species and the host immune response (Thomas et al., 2024). *Babesia*, a genus of protozoan parasites in the family Babesiidae, is primarily transmitted through tick bites. In wild animals, *Babesia* infections are often subclinical but can become pathogenic under certain conditions, such as when the parasite infects an unnatural host, the host is stressed due to captivity or immunosuppression, or there are changes in habitat quality or climate fluctuations. The clinical signs of *Babesia* spp. infection typically include fever, anemia, and hemoglobinuria (Alvarado-Rybak et al., 2016).

Haemoparasites have been reported in tiger populations in various countries, including Brazil, India, Italy, and the USA (André et al., 2012; Lewis et al., 2012; Pawar et al., 2012; Iatta et al., 2020; Cerreta et al., 2022; Grillini et al., 2023; Kolangath et al., 2024a). Most of these studies utilized molecular techniques, predominantly targeting the nuclear small subunit 18S rRNA gene (Pawar et al., 2012; Iatta et al., 2020; Grillini et al., 2023; Kolangath et al., 2024a). Molecular detection and characterization of these pathogens in wild animals provide valuable insights into their prevalence as well as related genetic data, which are crucial for understanding the potential risks the pathogens pose to both wildlife and domestic animals (Millán et al., 2016). However, data on haemoparasites in tigers are still relatively scarce in some regions, including in Thailand.

The aim of this study was to conduct a comprehensive molecular detection and characterization of haemoparasites in tigers housed at a wildlife breeding center in Thailand using advanced molecular techniques, including polymerase chain reaction (PCR) and sequencing methods.

## 2 Materials and methods

### 2.1 Blood sample collection

Blood samples were collected from 17 adult (6 males and 11 females) captive tigers at a wildlife breeding center in western Thailand in October 2019. Each tiger was housed in an individual cage, with the cages positioned next to one another. All tigers were asymptomatic at the time of sample collection. The blood samples were drawn from the saphenous vein under aseptic conditions and were kept in ethylenediamine tetraacetic acid (EDTA) tubes. These samples were obtained as part of a routine health check conducted by veterinarians as part of a collaboration programme between the Department of National Parks, Wildlife and Plant Conservation and the Monitoring and Surveillance Center for Zoonotic Diseases in Wildlife and Exotic Animals (MoZWE) of the Faculty of Veterinary Science, Mahidol University. This initiative aimed to assess the overall health of the tigers and screen for various infectious diseases, including those caused by tick-borne pathogens. After collection, the blood samples were stored at 4 °C and sent to MoZWE for further laboratory examination.

### 2.2 Examination and identification of ticks

The tigers were directly examined for the presence of ticks; the tigers’ entire bodies, including areas such as the head, ears, neck, back, abdomen, and feet, were examined while the animals were anesthetized for physical examination and blood collection. However, due to time constraints and the need to prioritize the safety of the animals, it was not possible to collect ticks from all the tigers or to collect all the ticks found on each tiger, which would have allowed the mean number of ticks per host to be calculated. Instead, we focused on collecting a representative sample to estimate the tick abundance. The collected ticks were carefully removed using fine-tipped forceps, preserved in 70% ethanol, and then sent to the Vector-Borne Diseases Research Unit at the Faculty of Veterinary Science, Mahidol University, for species identification. The identification was performed using standard taxonomic keys, including those outlined by Kohls (1957).

### 2.3 DNA extraction, multiplex polymerase chain reaction, and sequencing

Genomic DNA was extracted from blood samples using the Genomic DNA Mini Kit® (Geneaid, Taipei, Taiwan) according to the manufacturer’s protocol. Multiplex polymerase chain reaction (multiplex PCR) tests were conducted to detect *Ehrlichia canis*, *Hepatozoon* spp., and *Babesia* spp. infections. Three specific primer pairs were used for the multiplex PCR amplification of the VirB9 protein gene of *E. canis* (forward: Ehr1401F, 5′-CCA TAA GCA TAG CTG ATA ACC CTG TTA CAA-3′; reverse: Ehr1780R, 5′-TGG ATA ATA AAA CCG TAC TAT GTA TGC TAG-3′) with an expected PCR product consisting of 380 base pairs (Kledmanee et al., 2009); the 18S rRNA gene of *Hepatozoon* spp. (forward: Hep001F, 5′-CCT GGC TAT ACA TGA GCA AAT CTC AAC TT-3′; and reverse: Hep737R, 5′-CCA ACT GTC CCT ATC AAT CAT TAA AGC-3′) with an expected PCR product consisting of 737 base pairs (Kledmanee et al., 2009); and the 18S rRNA gene of *Babesia* spp. (forward: Ba143-167, 5′-CCG TGC TAA TTG TAG GGC TAA TAC A-3′; reverse, Ba694-667, 5′-GCT TGA AAC ACT CTA RTT TCT CAA AG-3′) with an expected PCR product consisting of 525 base pairs (Spolidorio et al., 2011). The total volume of the prepared multiplex PCR reaction mixture was 50 μl, consisting of 5 μl of template DNA (approximately 30–80 ng), 25 μl of 1 × multiplex PCR master mix (Qiagen, Hilden, Germany), and 1 μl of each primer at a final concentration of 0.2 μM. Amplification was performed using a C1000 Touch thermal cycler (BIO-RAD, Hercules, CA, USA). The thermocycling profile included an initial denaturation step at 95 °C for 15 min, followed by 35 cycles of denaturation at 94 °C for 45 s, annealing at 61 °C for 45 s, and extension at 72 °C for 1 min; a final extension step was then performed at 72 °C for 10 min.

The amplified PCR products were separated by electrophoresis on a 2.0% agarose gel stained with GelRed (Biotium, Fremont, CA, USA) and visualized using UV light. The GeneRuler™ 100 bp DNA ladder (Thermo Scientific, Vilnius, Lithuania) was used to estimate the size of the DNA fragments on the gel. PCR products were purified and sequenced in both directions on the ABI 3730XL DNA Analyzer using the services provided by U2Bio (Thailand) Co., Ltd., Bangkok, Thailand.

The chromatograms of both the VirB9 protein and 18S rRNA genes were examined. The forward and reverse sequences were manually trimmed, edited, and assembled to produce consensus sequences using BioEdit software version 7.2.5 (Hall, 1999). Sequence alignment was then performed using the ClustalW tool within the MEGA 11 software (Tamura et al., 2021). The newly generated haemoparasite consensus sequences were compared with sequences in the GenBank database using the Basic Local Alignment Search Tool (BLAST). Query coverage denotes the percentage of a sequence that aligns with a reference sequence in the GenBank database. The query coverage can be low if the sequence being investigated is short, the comparable sequences in GenBank are shorter, or there is significant divergence between the query sequence and all the available sequences in GenBank. Lower query coverage leads to fewer nucleotides being compared, which increases the likelihood of errors in the analysis. Therefore, to ensure accuracy, only DNA reference sequences from the GenBank database that achieved 100% query coverage were used for comparison with our haemoparasite sequences. Following the species identification using GenBank, the DNA sequences of the haemoparasite samples obtained in this study were deposited in GenBank; the accession numbers of these sequences are listed in Table 1, Table 2.

**Table 1:** VirB9 protein sequences of *Ehrlichia canis* detected in this study listed alongside the reference sequences downloaded from GenBank for use in the phylogenetic analysis.

Species	Host	Country of origin	GenBank ID
*Ehrlichia canis*	Dog (*Canis lupus familiaris*)	USA	AY205341
		USA	AY205342
		India	MN256130
		India	OL677476
		India	OL677477
		India	OP471415
		India	PP756431
		Thailand	PQ153228
	Tiger (*Panthera tigris*)	Thailand	PQ673868^a^
*Ehrlichia chaffeensis*	Cell culture	–	AF392617
*Anaplasma phagocytophilum*	Cell culture	–	AF392618

a Sequence generated in the present study.

**Table 2:** 18S rRNA gene sequences of the *Hepatozoon* species detected in this study listed alongside the reference sequences downloaded from GenBank for use in the phylogenetic analysis.

Species	Host	Country of origin	GenBank ID
*Hepatozoon canis*	Crab-eating fox (*Dusicyon thous*)	Brazil	AY461375
	Red fox (*Vulpes vulpes*)	Spain	AY731062
	Dog (*Canis lupus familiaris*)	Taiwan	EU289222
	Red fox (*Vulpes vulpes*)	Italy	KP715303
	Dog (*Canis lupus familiaris*)	Malaysia	KT267960
	Red fox (*Vulpes vulpes*)	Czech Republic	KU893125
	Dog (*Canis lupus familiaris*)	Czech Republic	KU893126
	Golden jackal (*Canis aureus*)	Austria	KX712123
	Golden jackal (*Canis aureus*)	Romania	KX712129
	Tick (*Haemaphysalis longicornis*)	Japan	LC169075
	Dog (*Canis lupus familiaris*)	India	MH922768
	Dog (*Canis lupus familiaris*)	Israel	MK091088
	Asiatic wild dog (*Cuon alpinus*)	Thailand	MK144332
	Tick (*Haemaphysalis longicornis*)	China	MT107087
	Dog (*Canis lupus familiaris*)	China	MT107091
	Raccoon (*Procyon lotor*)	Czech Republic	OQ816791
	Brown dog tick (*Rhipicephalus sanguineus*)	Turkey	OR661245
	Tiger (*Panthera tigris*)	Thailand	PQ666877^a^
*Hepatozoon felis*	Cat (*Felis catus*)	Spain	AY628681
	Bengal tiger (*Panthera tigris tigris*)	India	HQ829445
	Asiatic lion (*Panthera leo persica*)	India	KX017290
	Tick (*Rhipicephalus sanguineus*)	Thailand	KY056823
	Caracal (*Caracal caracal*)	South Africa	MK621305
	Tiger (*Panthera tigris*)	India	OL852087
	Asiatic lion (*Panthera leo persica*)	India	ON075470
	Eurasian lynx (*Lynx lynx*)	China	PP528683
	Tiger (*Panthera tigris*)	Thailand	PQ666876^a^
*Hepatozoon mariae*	Gidgee skink (*Egernia stokesii*)	Australia	KF992711
	Gidgee skink (*Egernia stokesii*)	Australia	KF992712
*Hepatozoon martis*	European pine marten (*Martes martes*)	Bosnia and Herzegovina	MG136687
	Beech marten (*Martes foina*)	Croatia	MG136688
*Hepatozoon procyonis*	South American coati (*Nasua nasua*)	Brazil	MF685390
	South American coati (*Nasua nasua*)	Brazil	MF685397
	South American coati (*Nasua nasua*)	Brazil	MF685400
*Hepatozoon ursi*	Black bear (*Ursus thibetanus japonicus*)	Japan	EU041718
	Sloth bear (*Melursus ursinus*)	India	HQ829429
*Adelina grylli*	House cricket (*Gryllus bimaculatus*)	–	DQ096836

a Sequences generated in the present study.

### 2.4 Phylogenetic analysis

To elucidate the evolutionary relationships between the haemoparasite sequences detected in this study, the reference species sequences, and the sequences of closely related species found in GenBank, a phylogenetic tree was constructed. This analysis utilized the maximum likelihood (ML) method and was performed entirely within MEGA 11. Based on the value of the Bayesian Information Criterion (BIC) calculated in MEGA, the most appropriate substitution models for the VirB9 protein gene and the 18S rRNA gene were identified as the Tamura 3-parameter (T92) and the Tamura 3-parameter plus gamma distribution (T92+G), respectively. The robustness of the tree branches was confirmed using 1000 bootstrap replicates. The reference sequences from GenBank used in the phylogenetic analysis of the VirB9 protein gene and the 18S rRNA gene are listed in Table 1, Table 2, respectively.

## 3 Results

### 3.1 Haemoparasite detection

According to the multiplex PCR results, haemoparasites were detected in the blood of four male and eight female tigers out of 17 tigers (70.6%). Of these, 10 tigers (58.8%) were infected with *E. canis*. Additionally, one tiger (5.9%) was co-infected with *Hepatozoon* sp. and *E. canis*, and another tiger (5.9%) was infected with *Hepatozoon* sp. only. No DNA of *Babesia* spp. was found in any of the analyzed blood samples.

The nucleotide sequences confirmed the presence of *E. canis* and identified the *Hepatozoon* species found in two tigers, as detailed in Table 3. The VirB9 protein gene sequences of *E. canis* (GenBank: PQ673868) matched 13 reference sequences of *E. canis* that had 100% query coverage with a similarity of 98.8–100%. Regarding the *Hepatozoon* species, two 18S rRNA gene sequences were analyzed: one sequence (GenBank: PQ666876) matched 79 reference sequences of *Hepatozoon felis* with 97.6–99.7% similarity, and the other sequence (from the co-infected tiger) (GenBank: PQ666877) matched 86 reference sequences of *Hepatozoon canis* with 98.1–99.9% similarity.

**Table 3:** BLAST results for *Ehrlichia canis* and *Hepatozoon* spp.

Species	GenBank ID	Species match	Similarity (%)
*Ehrlichia canis*	PQ673868	*Ehrlichia canis*	98.8–100 (*n* = 13)
*Hepatozoon* sp.	PQ666876	*Hepatozoon felis*	97.6–99.7 (*n* = 79)
*Hepatozoon* sp.	PQ666877	*Hepatozoon canis*	98.1–99.9 (*n* = 86)

*Note:* Only DNA reference sequences from the GenBank database that achieved 100% query coverage were used for comparison with our haemoparasite sequences; “*n*” denotes the number of reference sequences.

### 3.2 Tick identification

**Fig. 1:**
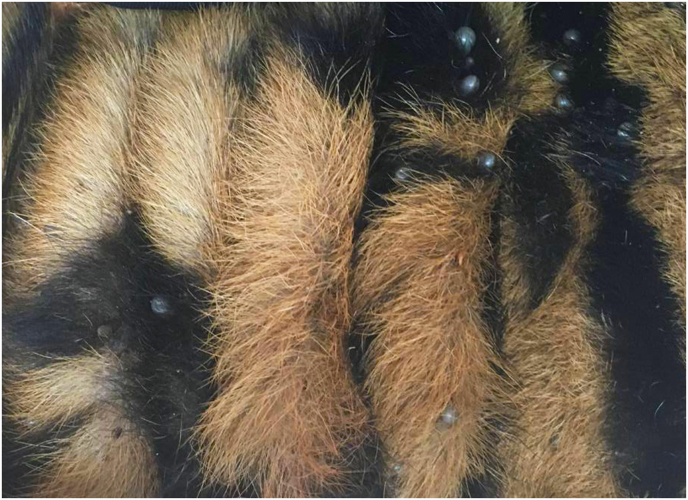
The ticks *Rhipicephalus sanguineus* infesting tiger’s neck.

**Fig. 2:**
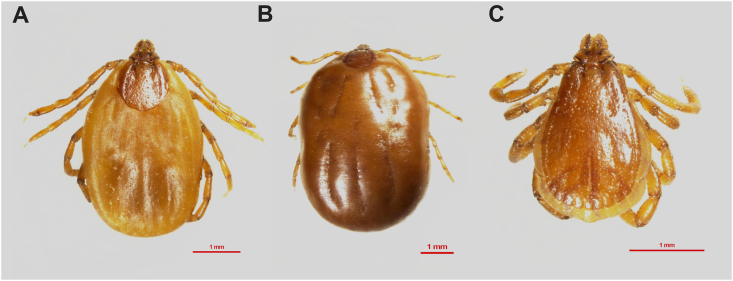
The brown dog tick *Rhipicephalus sanguineus*. **A** Unengorged female, dorsal view. **B** Engorged female, dorsal view. **C** Male, dorsal view.

A total of 58 ticks were collected from infested tigers: 43 of these ticks were adult females (30 engorged and 13 unengorged), and 15 were adult males. The majority of the ticks were obtained from the neck area (Fig. 1), with the back being the second most common collection site. All ticks were morphologically identified as brown dog ticks (*Rhipicephalus sanguineus*) (Fig. 2).

### 3.3 Phylogenetic analyses

**Fig. 3:**
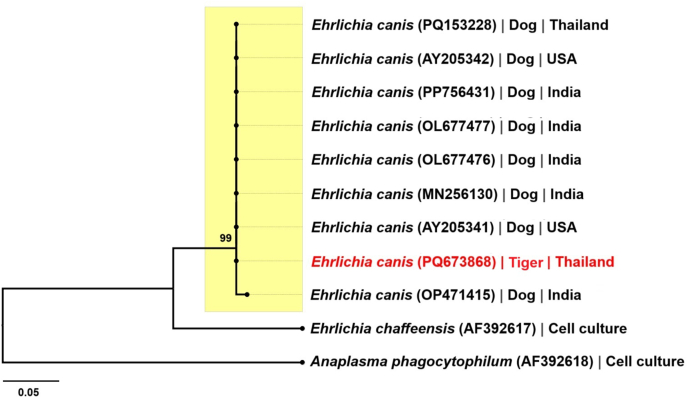
Phylogenetic tree of *Ehrlichia canis* inferred from maximum likelihood (ML) analysis of the VirB9 protein gene (with bootstrap values defined for 1000 replicates). Node labels indicate bootstrap values > 90%. The sequence of *E. canis* detected in this study is indicated in red. Where available, the host and country of origin are given for each species sequence.

**Fig. 4:**
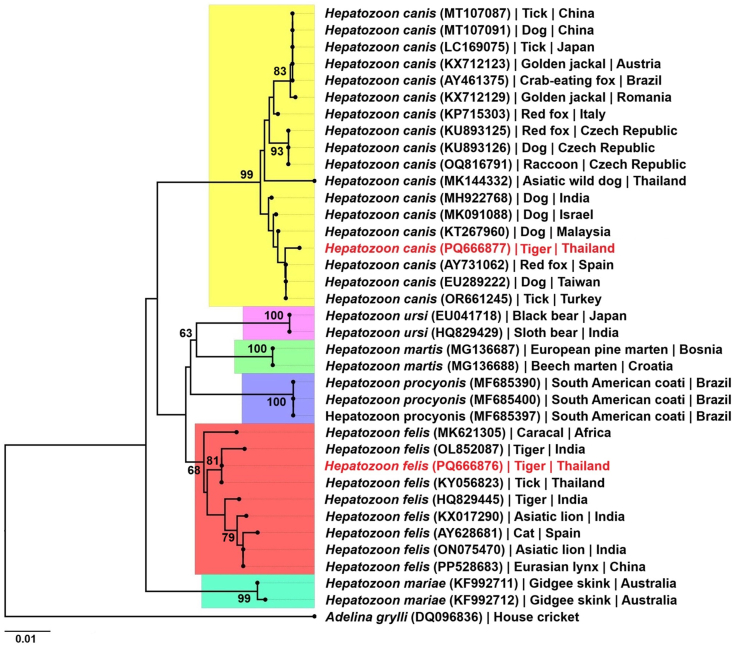
Phylogenetic tree of *Hepatozoon* species inferred from maximum likelihood (ML) analysis of the 18S rRNA gene (with bootstrap values defined for 1000 replicates). Node labels indicate bootstrap values > 60%. The sequences of *Hepatozoon* spp. detected in this study are indicated in red. The host and country of origin are given for each species sequence.

The phylogenetic analyses performed in this study used the newly generated *E. canis* sequence (GenBank: PQ673868) and two *Hepatozoon* spp. sequences (GenBank: PQ666876 and PQ666877), along with numerous reference sequences from GenBank; details of the results of these analyses are given in Fig. 3, Fig. 4. The new *E. canis* sequence clustered with those from the database in the phylogenetic tree derived from the ML analysis of VirB9 protein gene sequences of *E. canis* (Fig. 3), confirming its identification as *E. canis*.

The phylogenetic tree of the 18S rRNA gene sequences from various *Hepatozoon* spp. revealed distinct clustering patterns (Fig. 4). The sequence PQ666876 was grouped within the *H. felis* cluster, confirming its identification as *H. felis*, whereas the sequence PQ666877 was grouped within the *H. canis* cluster, confirming its identification as *H. canis*. Both clusters were clearly differentiated.

## 4 Discussion

Globally, reports of haemoparasite infections caused by bacteria and protozoans in tigers are scarce. To the best of our knowledge, this study is the first in Thailand to explore the occurrence of these haemoparasites. We found a haemoparasite infection rate of 70.6% among the examined captive tiger population, which was significantly higher than the rate of 46.15% observed in wild tigers in the Vidarbha region of Maharashtra, India (Kolangath et al., 2024a). Lower infection rates have also been reported in other captive settings, including a wildlife safari park in Italy, where all the animals tested positive for pathogens; the haemoparasite infection rate was found to be 55% (Iatta et al., 2020). Elevated infection rates in captive tigers can be attributed to increased exposure to ticks and other insect vectors, particularly when enclosures are sited close to natural habitats with robust vector populations. Additionally, the close proximity of animals in captivity can facilitate the spread of vectors such as ticks, further increasing the animals’ exposure to haemoparasites (Iatta et al., 2020; Hrnková et al., 2021; Cerreta et al., 2022). In the present study, the tigers were housed in close proximity, allowing tick movement between them, potentially leading to the transmission of parasites from infected tigers to non-infected tigers. The parasites detected in captive tigers included *E. canis*, *H. felis*, and *H. canis*, all of which are transmitted by the tick *R. sanguineus* (Baneth et al., 2001; Fourie et al., 2013; Bhusri et al., 2017).

In this study, 58.8% of the tigers were found to be infected with *E. canis*. Previous research has documented *E. canis* infections in various captive felids in Brazilian Zoos, including jaguars (*Panthera onca*), ocelots (*Leopardus pardalis*), jaguarundis (*Puma yagouaroundi*), margays (*Leopardus wiedii*), and little spotted cats (*Leopardus tigrinus*) (André et al., 2010; Hrnková et al., 2021). Another study reported the presence of *E. canis* in various Brazilian captive wild felids, such as pumas (*Puma concolor*) and lions (*Panthera leo*), and also detected *E. chaffeensis* infections in tigers (*Panthera tigris*) (André et al., 2012). Our study is the first to report *E. canis* infection in tigers using the PCR method. This identification was substantiated by comparing our VirB9 protein gene sequence with available *E. canis* sequences in the GenBank database. The phylogenetic tree analysis revealed no genetic variation in *E. canis* infection across different hosts (dog and tiger), suggesting that this pathogen does not undergo significant genetic changes in different host species. Despite the known influence of host-pathogen interactions on genetic diversity (Sironi et al., 2015) no such variations were found in the present study; this may have been because only one gene was analyzed. Therefore, further studies involving comparative genomic analyses and larger sample sizes are necessary to thoroughly understand this phenomenon.

The PCR analysis conducted here detected *Hepatozoon* spp. infections in two tigers. Species-level identification through comparison with GenBank sequences identified these infections as *H. canis* and *H. felis*. Depending on the species, these parasites are known to infect a diverse range of hosts, including mammals, birds, reptiles, and amphibians (Smith, 1996). *Hepatozoon canis* is a widespread tick-borne protozoan that infects dogs and cats. A previous study in Bangkok, Thailand, found that 11.4% of the dogs and 32.3% of the cats tested were infected with *H. canis* (Jittapalapong et al., 2006). This parasite also has a high prevalence in foxes across Europe (Cardoso et al., 2014) and has been detected in captive tigers and other captive felid species in Italy (Iatta et al., 2020). Our findings align with those of previous reports, confirming that tigers can host *H. canis*. Similarly, *H. felis* has been reported in tigers at several locations, including the Nehru Zoological Park in Hyderabad, India (Pawar et al., 2012), the Vidarbha region of Maharashtra State, India (Kolangath et al., 2024a), and the Tadoba Tiger Reserve, India (Kolangath et al., 2024b). There have also been reports of *H. felis* infections in both domestic and wild cats in various countries (Metzger et al., 2008; Salakij et al., 2010; Pawar et al., 2012). Our phylogenetic analysis of the 18S rRNA gene indicated clear clustering among the *Hepatozoon* species but no host-specific relationships within species, thus supporting previous findings that these parasites are not host-specific and can infect both domestic and wild felids and canids around the world (Pawar et al., 2012).

All ticks collected from the infested tigers were morphologically identified as *R. sanguineus*. This tick species is recognized as a significant vector for *E. canis*, *H. felis*, and *H. canis* (Groves et al., 1975; Baneth et al., 2007; Fourie et al., 2013; Bhusri et al., 2017). In Thailand, *H. felis* has been detected in *R. sanguineus* ticks collected from captive lions (Bhusri et al., 2017). Unfortunately, the scope of this study was limited as no ticks were tested for these pathogens. However, the infection of this tick species with *E. canis*, *H. felis*, or *H. canis* remains a possibility, given that only *R. sanguineus* was found on the infected tigers. This tick species has a three-host life cycle, with each developmental stage (larva, nymph, and adult) potentially choosing a different host (Dantas-Torres, 2010). This allows the ticks to acquire or transmit pathogens with each blood meal. The dynamics of tick-borne disease transmission is heavily influenced by the proximity of diverse vertebrate hosts, wherein overlapping ecological niches among different species facilitate the expansion of the parasites’ geographical distribution, abundance, and host diversity (André, 2018). Such conditions make a wildlife breeding center an ideal environment for *R. sanguineus* ticks and their reservoir hosts. This scenario may mirror outbreaks that have been observed in several zoological gardens that were potentially initiated by freely roaming dogs and cats that commonly carry local ticks such as *R. sanguineus*. These domestic dogs and cats can significantly contribute to an increase in the population of infected ticks within areas where they frequently roam (Hrnková et al., 2021).

## 5 Conclusions

This study identified infections of three haemoparasites, *E. canis*, *H. felis*, and *H. canis*, in captive tigers at a wildlife center in Thailand. The detection of these parasites highlights the complex interactions present within captive environments and elucidates the dynamics of disease transmission, emphasizing the need for preventive measures. Therefore, in such centers, steps should be taken to control ectoparasites and to manage domestic animals in ways that reduce the risk of infection.

## CRediT authorship contribution statement

**Tanasak Changbunjong:** Conceptualization, Methodology, Formal analysis, Investigation, Writing – original draft, Writing – review & editing, Visualization, Supervision. **Tatiyanuch Chamsai:** Conceptualization, Methodology, Investigation, Writing – original draft, Writing – review & editing. **Siriporn Tangsudjai:** Conceptualization, Methodology, Investigation, Writing – review & editing. **Nareerat Sangkachai:** Investigation, Writing – review & editing. **Chalisa Mongkolphan:** Investigation, Writing – review & editing. **Luxsana Prasittichai:** Methodology, Investigation, Writing – review & editing. **Tanawat Chaiphongpachara:** Conceptualization, Formal analysis, Writing – original draft, Writing – review & editing, Visualization, Supervision.

## Ethical approval

The study received ethical approval from the Animal Care and Use Committee of the Faculty of Veterinary Science at Mahidol University, Thailand (Ref. MUVS-2019-02-07).

## Data availability

The data supporting the conclusions of this article are included within the article. The newly generated sequences are deposited in the GenBank database under the accession numbers PQ673868 (VirB9 protein gene), and PQ666876 and PQ666877 (18S rRNA gene).

## Funding

This research was funded by the Monitoring and Surveillance Center for Zoonotic Diseases in Wildlife and Exotic Animals (MoZWE), Faculty of Veterinary Science, Mahidol University.

## Declaration of competing interests

The authors declare that they have no known competing financial interests or personal relationships that could have appeared to influence the work reported in this paper.
